# Coronary Artery Inflammation and Epicardial Adipose Tissue Volume in Relation with Atrial Fibrillation Development

**DOI:** 10.3390/diagnostics15162003

**Published:** 2025-08-11

**Authors:** Renáta Gerculy, Imre Benedek, István Kovács, Nóra Rat, Ioana-Patricia Rodean, Botond Barna Mátyás, Emanuel Blîndu, Delia Păcurar, Ciprian-Gelu Grigoroaea, Theodora Benedek

**Affiliations:** 1 Department of Cardiology, “George Emil Palade” University of Medicine, Pharmacy, Science and Technology of Târgu Mureș, 540139 Târgu Mureș, Romania; gerculy_renata@yahoo.com (R.G.); imrebenedek@yahoo.com (I.B.); rat.nora@umfst.ro (N.R.); ioana.rodean@umfst.ro (I.-P.R.); emi.blindu@yahoo.com (E.B.); theodora.benedek@umfst.ro (T.B.); 2 Doctoral School of Medicine and Pharmacy, “George Emil Palade” University of Medicine, Pharmacy, Science and Technology of Târgu Mureș, 540139 Târgu Mureș, Romania; matyas_botond@yahoo.com; 3 Clinic of Cardiology, Mureș County Emergency Clinical Hospital, 540136 Târgu Mureș, Romania; deliapacurar99@gmail.com (D.P.); ciprian_grig@yahoo.com (C.-G.G.); 4 Center of Advanced Research in Multimodality Cardiac Imaging, CardioMed Medical Center, 540124 Târgu Mureș, Romania

**Keywords:** atrial fibrillation, epicardial adipose tissue, pericoronary inflammation, coronary computed tomography, fat attenuation index, left atrial volume index, CAD-RADS classification

## Abstract

**Background/Objectives:** Atrial fibrillation (AF) is associated with increased epicardial adipose tissue (EAT), atrial dilation, and coronary inflammation, though causality remains unclear. Cardiac computed tomography (CCT) allows for precise quantification of EAT volume and the left atrial volume index (LAVI), along with the calculation of the fat attenuation index (FAI), indicating coronary inflammation. Combined with the Coronary Artery Disease-Reporting and Data System (CAD-RADS), these imaging markers may improve AF risk stratification. This study evaluates the association between peri-atrial EAT volumes, LAVI, CAD-RADS, and FAI scores in AF patients using advanced AI platforms. **Methods:** This retrospective study analyzed 122 patients presenting with angina-type pain and a low-to-intermediate likelihood of CAD, who underwent CCT. Patients were divided into two groups based on rhythm status: 42 with AF and 80 without AF. Total EAT, left atrial (LA-EAT), and bi-atrial EAT (BA-EAT) volumes were assessed, along with LAV, CAD-RADS classification, and FAI scores measured using CaRi-Heart^®^ and syngo.via Frontier^®^. **Results:** AF patients exhibited significantly higher EAT volumes (total EAT: 231.8 ± 45.85 vs. 153.2 ± 54.14 mL, *p* < 0.0001; LA-EAT: 23.55 ± 6.44 vs. 15.54 ± 8.49 mL, *p* < 0.0001; BA-EAT: 50.24 ± 12.69 vs. 39.84 ± 15.70 mL, *p* = 0.0002) and elevated LAVI values (57.7 ± 11.44 vs. 45.9 ± 12.58 mL/m^2^, *p* < 0.0001). ROC analyses confirmed strong diagnostic performance of total EAT (AUC = 0.869), LA-EAT (AUC = 0.776), BA-EAT (AUC = 0.703), and the LAVI (AUC = 0.756). Higher CAD-RADS categories (2–5) were more frequent in AF, although significant differences were observed only in the lowest category (0–1; 26.2% AF vs. 47.8% non-AF, *p* = 0.032). Total FAI scores were also higher in AF patients (14.83 ± 10.16 vs. 12.37 ± 7.89, *p* = 0.044). **Conclusions:** Increased EAT volumes, an elevated LAVI, and higher FAI scores are significantly associated with AF, suggesting a combined structural and inflammatory substrate. EAT, the LAVI, the FAI, and CAD-RADS collectively represent valuable non-invasive imaging biomarkers for early AF risk assessment.

## 1. Introduction

Multiple studies have demonstrated a clear relationship between epicardial adipose tissue (EAT) and atrial fibrillation (AF) [1,2]. More specifically, research has identified a significant correlation between the volume of adipose tissue surrounding the atria and the occurrence of AF [3,4,5]. EAT, a metabolically active fat depot encompassing the heart, includes distinct regions such as peri-atrial and pericoronary fat, both of which have been associated with various cardiovascular conditions, notably AF [6]. Recent advancements in cardiac computed tomography (CCT) allow for the accurate quantification of EAT volume and the assessment of its inflammatory activity via the fat attenuation index (FAI), a marker of tissue inflammation [7]. Growing evidence indicates that both the extent of EAT and its inflammatory characteristics play crucial roles in the onset and progression of AF [8].

Pericoronary inflammation, assessed through the FAI using CCT, provides valuable insights into the inflammatory activity of adipose tissue adjacent to coronary arteries. Several studies have documented strong associations between increased pericoronary fat attenuation—particularly around the left circumflex artery—and higher rates of both the incidence and recurrence of AF [9,10,11]. These findings suggest that increased pericoronary inflammation could represent a more widespread inflammatory state, potentially contributing to arrhythmogenic conditions and recurrent AF episodes [12,13].

In parallel, left atrial enlargement has long been recognized as a key structural predictor of AF. CCT–derived measurements such as left atrial volume (LAV) and the left atrial volume index (LAVI) have shown strong and independent associations with AF detection, severity, and recurrence. CCT–LAVI has been demonstrated to outperform echocardiographic measurements in predicting AF recurrence after ablation or cardioversion, likely due to its superior spatial resolution and reproducibility [14,15,16]. For instance, each 1 mL/m^2^ increase in LAVI score was associated with a 6% greater risk of AF recurrence following cardioversion [14], and the LAVI measured by CCT predicted AF recurrence with significantly higher values in recurrent cases compared to non-recurrent ones [15]. Furthermore, the LAVI has been identified as an independent predictor of long-term rhythm outcomes following catheter ablation [16].

Integrating EAT and LAVI assessment with the Coronary Artery Disease-Reporting and Data System (CAD-RADS) framework may further enhance the prognostic utility of CCT scans. The CAD-RADS is a structured scoring system designed to classify CAD severity, and evidence indicates potential synergies when combined with EAT parameters [17]. Higher CAD-RADS scores, indicative of more advanced coronary lesions, have been associated with greater EAT volumes and increased pericoronary inflammation [18]. This integrated approach suggests that patients with elevated CAD-RADS classifications might concurrently exhibit significant epicardial inflammatory activity and atrial enlargement, placing them at increased risk for AF.

Mechanistically, inflammation within peri-atrial and pericoronary EAT compartments results in the secretion of pro-inflammatory cytokines and chemokines, such as interleukin-1β and tumor necrosis factor-α. These mediators contribute to myocardial fibrosis, atrial dilation, and electrophysiological remodeling, providing a biological basis for the observed clinical correlations between EAT characteristics, atrial enlargement, and AF [19]. This biochemical interplay underpins the rationale for combining structural (LAVI), adipose (EAT), and inflammatory (FAI) imaging markers in a multidimensional strategy for AF risk evaluation.

Our study investigates the associations among EAT, the LAV/LAVI, pericoronary inflammation, CAD severity, and AF. The primary objective was to determine whether increased volumes of peri-atrial EAT and atrial enlargement correlate with AF occurrence. Additionally, we examined the relationship between CAD-RADS classifications and FAI scores using advanced AI-driven imaging platforms, including CaRi-Heart^®^ (Caristo Diagnostics, Oxford, UK) and syngo.via Frontier^®^ (Siemens Healthineers, Erlangen, Germany).

## 2. Materials and Methods

### 2.1. Study Design and Population

Our retrospective, observational, single-center study included 122 adult patients with a low-to-intermediate likelihood of CAD who underwent CCT between 2022 and 2024 for the evaluation of angina-like symptoms. Among these, 42 patients had a confirmed history of AF. Initially, 203 AF patients were identified; however, 161 were excluded based on the following criteria: uncontrolled AF with a heart rate > 100–120 bpm (*n* = 38), significant renal dysfunction with eGFR < 30–45 mL/min/1.73 m^2^ (*n* = 26), known allergy to iodinated contrast agents (*n* = 9), prior AF ablation (*n* = 34), presence of intracardiac thrombus (*n* = 12), severe obesity with BMI > 40–45 (*n* = 17), inability to perform an adequate breath-hold (*n* = 10), and poor CT image quality due to motion artifacts or incomplete contrast opacification (*n* = 15).

For the control group, over 1000 patients without a history of arrhythmia underwent CCT during the same period. From this larger cohort, 80 individuals were selected and matched to the AF group based on age, gender, and cardiovascular risk profiles to ensure comparability. All included patients were in sinus rhythm or had a rate-controlled AF at the time of scanning, allowing for high-quality ECG-gated image acquisition. Each CCT study included both non-contrast and contrast-enhanced phases, enabling the comprehensive evaluation of coronary artery calcium score (CACs), CAD-RADS classification, pericoronary FAI, EAT volume, and LAVI score.

**Figure 1:**
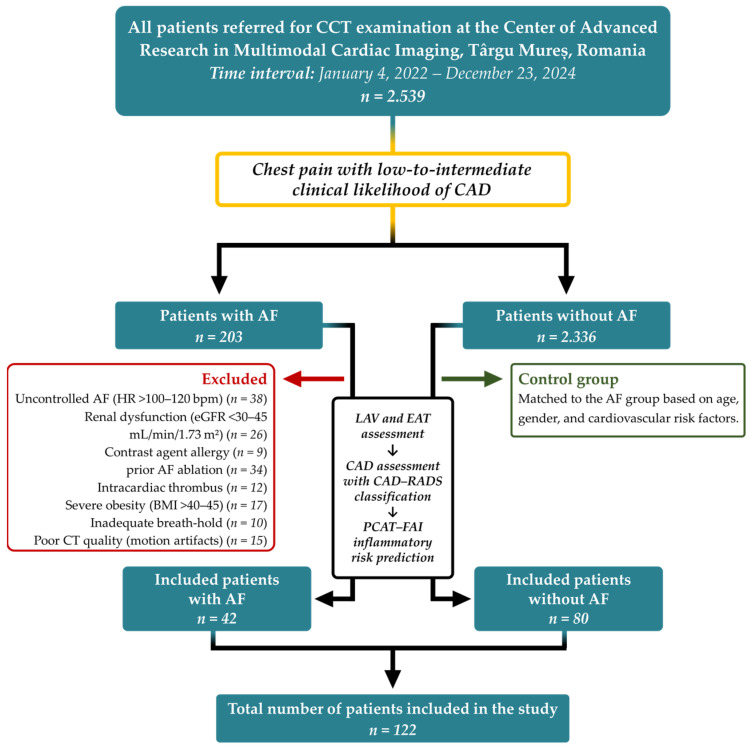
Flow diagram illustrating the study design and patient selection process. Abbreviations: LAV–left atrial volume; EAT–epicardial adipose tissue; CAD–coronary artery disease; CAD-RADS–Coronary Artery Disease-Reporting and Data System; PCAT–pericoronary adipose tissue; FAI–fat attenuation index.

Figure 1 illustrates the study design, patient selection process, and grouping into AF and non-AF cohorts, including inclusion and exclusion criteria, as well as the subsequent imaging and analysis workflow.

### 2.2. CCT Acquisition Protocol

All of the subjects included in the study underwent CCT using a 128-slice CT (Siemens Somatom Definition AS, Siemens Healthcare, Erlangen, Germany) between 2022 and 2024 at the Center of Advanced Research in Multimodal Cardiac Imaging, Târgu Mureș, Romania. Scans were ECG-gated in patients with heart rates below 70 bpm, with beta-blockers or nitrates administered as needed. The protocol included a scan with zero contrast calcium scoring, which was followed by contrast-enhanced imaging using 80–100 mL of iodine-based contrast (adjusted to body weight) and a 50 mL saline flush at 5.5–6 mL/s during an inspiratory breath-hold. Images were archived electronically and forwarded to Caristo Diagnostics (Oxford, UK) for analysis of the pericoronary adipose tissue fat attenuation index (PCAT-FAI); results were then returned and stored for further evaluation.

### 2.3. CAD, EAT Volume, and LAV Assessment

In the non-contrast (native) scans, we analyzed the Agatson score or coronary artery calcium score (CACs), while the contrast-enhanced scans were used to assess the degree of coronary artery stenosis based on the Coronary Artery Disease-Reporting and Data System (CAD-RADS). The CAD-RADS classification ranges from 0 to 5 according to the severity of stenosis: CAD-RADS 0 (0% narrowing), CAD-RADS 1 (1–24%), CAD-RADS 2 (25–49%), CAD-RADS 3 (50–69%), CAD-RADS 4 (70–99%), and CAD-RADS 5 (indicates 100% occlusion of the analyzed vessel).

EAT volume was also assessed using the native CCT scans. EAT was defined as the adipose tissue located between the myocardial surface and the visceral layer of the pericardium. Using dedicated post-processing software, EAT was identified based on its characteristic attenuation values, typically ranging from −190 to −30 Hounsfield Units (HU). The pericardial contour was manually or semi-automatically traced from the level of the pulmonary artery bifurcation to the apex of the heart in axial slices. The software then calculated total EAT volume by summing the cross-sectional fat areas within the defined pericardial boundary across all slices. Final EAT volume was expressed in milliliters (mL).

LAV was measured from contrast-enhanced CCT images using the biplane area-length method, following standard cardiac imaging protocols. Measurements were obtained in both axial and coronal planes at end-ventricular diastole, and the maximum LAV was recorded. The LAVI was then calculated by dividing LAV by body surface area (BSA) and it was expressed in mL/m^2^. This method is in line with previously validated approaches for CT-based atrial volume quantification [20].

### 2.4. Pericoronary Inflammation Assessment

Advanced techniques based on CCT allow for the quantification of attenuation gradients emerging from the outer surface of the coronary arteries, a region that is highly sensitive to inflammatory signaling that often leads to perivascular edema. Under normal physiological conditions, adipose tissue on CCT exhibits highly negative attenuation values, typically approaching −190 HU. During inflammation, changes in adipocyte morphology—marked by decreased cell size and lipid content—lead to a shift in attenuation values toward less negative levels, approaching approximately −30 HU. This transition reflects a compositional change from lipid-rich to more aqueous tissue, serving as a non-invasive imaging biomarker of vascular inflammation.

We evaluated two distinct parameters to characterize PCAT. First, we utilized the fat attenuation index (FAI-HU), expressed in HU, which provides a direct, unadjusted measure of local pericoronary inflammation. Subsequently, the FAI Score was introduced as an individualized measure of coronary inflammation across the three main epicardial arteries, adjusted for age and sex, allowing for a more personalized assessment of vascular inflammation risk.

**Figure 2:**
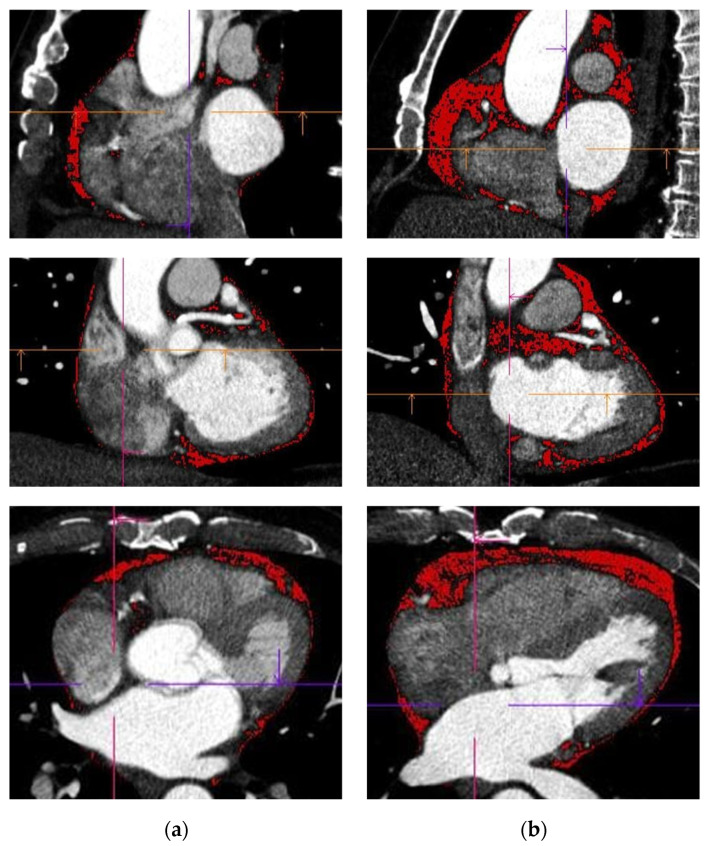
Imaging of the heart with quantification of EAT, marked by a red contour. Two representative cases from the study are shown: (**a**) a patient without AF, displaying a relatively low volume of EAT; (**b**) a patient with AF, showing a noticeably larger amount of EAT.

Figure 2 shows representative CCT images with quantification of EAT, highlighted by a red contour, comparing a patient without AF and low EAT volume to a patient with AF and markedly increased EAT volume.

### 2.5. Statistical Analysis

PCAT-FAI and FAI score were quantified for the three major coronary arteries (LAD, LCX, and RCA), alongside assessments of EAT volume and coronary artery stenosis using the CAD-RADS classification. All data were compiled in a Microsoft Excel database (Office 2019, Microsoft Corp., Redmond, WA, USA). The study analyzed 122 patients—42 with documented AF and 80 without—evaluating a total of 369 coronary arteries (123 LADs, 123 LCXs, and 123 RCAs). Categorical variables (e.g., AF status, CACs categories, CAD-RADS categories) were presented as counts and percentages, with group comparisons performed using the Chi-square or Fisher’s exact test. Continuous variables (e.g., FAI-HU, FAI Score, EAT volume, CACs) were expressed as mean ± standard deviation (SD) and compared using either the Mann–Whitney U test or the unpaired Student’s *t*-test, depending on the distribution of the data. ROC curve analysis was performed using MedCalc v20.2.18 (MedCalc Software Ltd., Ostend, Belgium), while uni- and multivariable analyses were performed with GraphPad Prism 10.3.1 (GraphPad Software, San Diego, CA, USA).

## 3. Results

### 3.1. Baseline Characteristics

Analyzing our study cohort, the subjects mean age did not differ significantly between the two groups (64.05 ± 7.84 vs. 63.53 ± 7.78 years, *p* = 0.66), indicating a relatively balanced distribution. The gender distribution was also similar, with 61.47% of the total population being male. No significant differences were found regarding body mass index (BMI), as the values were comparable between the groups (28.25 ± 5.07 vs. 27.05 ± 3.86, *p* = 0.256). As shown in Table 1, there were no statistically significant differences in cardiovascular risk factors between patients with AF and those without AF. The prevalence of hypertension was similar (29 [69.05%] vs. 46 [57.50%], *p* = 0.244), as was the presence of hypercholesterolemia (17 [40.48%] vs. 26 [32.50%], *p* = 0.42) and diabetes mellitus (10 [23.81%] vs. 22 [27.50%], *p* = 0.82). Tobacco use was also comparable between the two groups (11 [26.19%] vs. 16 [20.00%], *p* = 0.49), with no statistically significant differences observed.

Advanced techniques based on CCT allow for the quantification of attenuation gradients emerging from the outer surface of the coronary arteries, a region that is highly sensitive to inflammatory signaling that often leads to perivascular edema. Under normal physiological conditions, adipose tissue on CCT exhibits highly negative attenuation values, typically approaching −190 HU. During inflammation, changes in adipocyte morphology—marked by decreased cell size and lipid content—lead to a shift in attenuation values toward less negative levels, approaching approximately −30 HU. This transition reflects a compositional change from lipid-rich to more aqueous tissue, serving as a non-invasive imaging biomarker of vascular inflammation.

### 3.2. Imaging Biomarkers of Atrial and Coronary Pathology: LAVI, EAT, CACs, and CAD-RADS

CT-derived LAVs and LAVI scores were significantly higher in patients with AF compared to those without AF. The mean LAV was 103.3 ± 20.34 mL in the AF group versus 86.3 ± 22.94 mL in the non-AF group (*p* < 0.0001), while the LAVI score was also markedly elevated in the AF cohort (57.7 ± 11.44 vs. 45.9 ± 12.58 mL/m^2^, *p* < 0.0001). These findings underscore the structural remodeling associated with AF and reinforce the diagnostic value of CT-derived atrial volumetric parameters in arrhythmia risk stratification (Table 2).

We found statistically significant differences in EAT volume between the two groups. Patients with AF had a significantly higher total EAT volume compared to those without AF (231.8 ± 45.85 vs. 153.2 ± 54.14, *p* < 0.0001). When analyzing the left atrial EAT volume separately, it was also significantly higher in the AF group (23.55 ± 6.44 vs. 15.54 ± 8.49, *p* < 0.0001). Similarly, the bi-atrial EAT volume was significantly greater in Group 1 (patients with AF) compared to those without AF (50.24 ± 12.69 vs. 39.84 ± 15.70, *p* = 0.0002).

To explore the relationship between systemic obesity and EAT accumulation, we conducted a regression analysis comparing BMI and EAT volume in both groups. In the AF group, a statistically significant but weak positive correlation was observed (r = 0.1296, *p* = 0.0192), whereas in the non-AF group, no significant correlation was found (r = 0.0039, *p* = 0.5839).

The CACs was notably higher in the control group, consisting of patients without AF. In this group, CACss were significantly higher compared to those with AF (231.9 ± 76.98 vs. 455.1 ± 91.27, *p* < 0.0001). However, when the extent of CACss was categorized into three groups (<10, 10–400, and >400), no statistically significant differences were observed between the two study groups, as shown in Table 2.

We also compared the two study groups based on CAD-RADS categories. The CAD-RADS 0–1 category was more prevalent among patients without AF; however, the difference did not reach statistical significance (11 [26.19%] vs. 38 [47.75%], *p* = 0.0320). The distribution of CAD-RADS 2–3 was similar between the groups (17 [40.48%] vs. 25 [31.25%], *p* = 0.323), as was the distribution of CAD-RADS 4–5 (14 [33.33%] vs. 17 [21.25%], *p* = 0.189).

### 3.3. LAVI, EAT Volumes, and the Risk of AF

**Figure 3:**
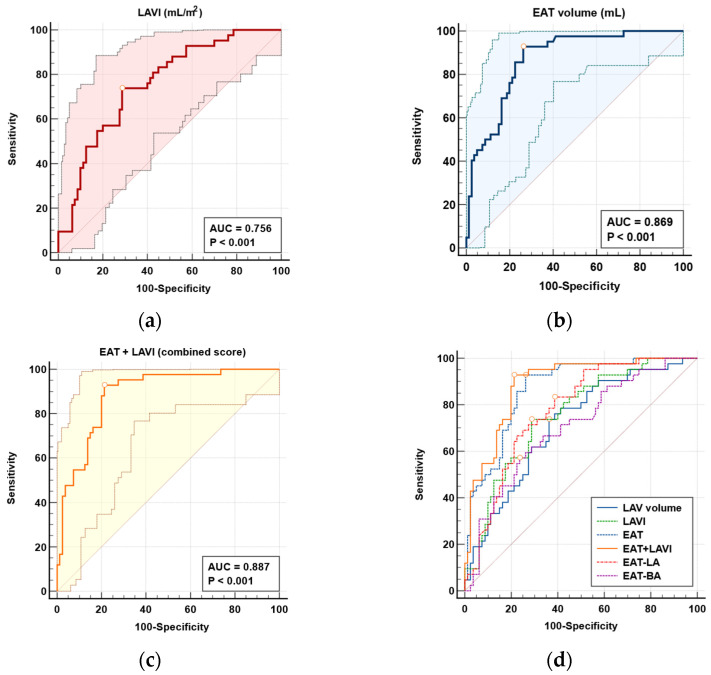
ROC curve analysis evaluating the diagnostic performance of EAT parameters and left atrial structural markers for predicting AF using CCT. (**a**) LAVI–Left Atrial Volume Index (red line, light red background area); (**b**) Total EAT–Epicardial Adipose Tissue (dark blue line, light blue background area); (**c**) combined score of EAT and LAVI (orange line, yellow background area); (**d**) comparison of all ROC curves demonstrating the relative predictive accuracy of each parameter in identifying AF, with each curve represented by a different color: LAV volume (blue), LAVI (green dotted), EAT (blue dotted), EAT + LAVI (orange), EAT-LA (pink dotted), and EAT-BA (purple dotted). Dots on each ROC curve indicate the optimal cutoff points determined by the Youden index.

ROC curve analysis highlighted the predictive value of left atrial volumetric markers measured by CCT. The LAVI demonstrated an AUC of 0.756 and LAV had an AUC of 0.713 (both *p* < 0.0001), indicating moderate diagnostic performance for AF. In comparison, EAT parameters showed stronger associations: total EAT volume had the highest predictive value with an AUC of 0.869 (*p* < 0.0001), followed by EAT surrounding the left atrium (EAT-LA) with an AUC of 0.776 (*p* < 0.0001), and bi-atrial EAT (EAT-BA) with an AUC of 0.703 (*p* < 0.0001). Among all parameters, total EAT demonstrated the highest discriminatory capacity. The LAVI and EAT-LA showed similar moderate predictive performance, while LAV and EAT-BA were slightly less effective. The analysis of optimal cutoff values indicated that total EAT achieved superior sensitivity and specificity compared to both localized EAT and atrial volume measures. Importantly, a combined score integrating total EAT and the LAVI further increased diagnostic accuracy, with an AUC of 0.887, sensitivity of 92.86%, and specificity of 78.75% (*p* < 0.0001). However, statistical comparison of ROC curves using the DeLong test showed that this improvement over EAT alone was not significant (*p* = 0.186), indicating that the added value of the combined score, while numerically higher, was modest. These findings suggest that total EAT is the most robust individual marker, while the LAVI offers valuable complementary structural information for identifying patients who are at risk of AF (Figure 3, Table 3).

### 3.4. CACs, FAI-Score, and CaRi-Heart^®^ Risk Score in AF and Non-AF Patients

**Figure 4:**
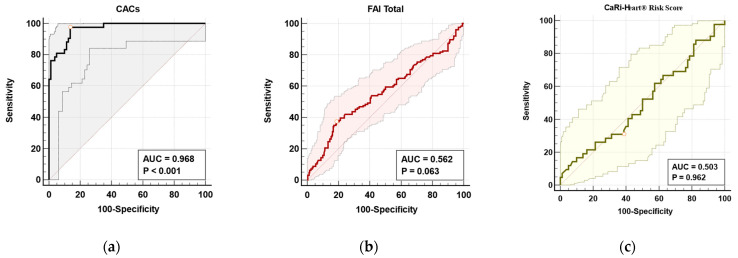
ROC analysis to evaluate the prognostic value of (**a**) CACs (black line, grey background area), (**b**) FAI score (red line, light red background area), and (**c**) CaRi-Heart^®^ Risk Score (olive green line, light yellow background area) in differentiating AF and non-AF patients. Dots on each ROC curve indicate the optimal cutoff points determined by the Youden index. Abbreviations: CACs–coronary artery calcium score; FAI–fat attenuation index; ROC–Receiver Operating Characteristic.

As shown in Figure 4, the prognostic value of the CACs was evaluated using ROC curve analysis. The AUC was 0.968 (*p* < 0.001), indicating excellent discriminatory capacity. At the optimal cutoff value (≤352.21), the model achieved a sensitivity of 97.6% and a specificity of 86.2%, reflecting high accuracy in predicting clinical outcomes (Figure 4a). In contrast, ROC analysis for the total FAI score yielded a lower AUC of 0.562 (*p* = 0.063), suggesting limited predictive performance and borderline statistical significance (Figure 4b). Furthermore, the CaRi-Heart^®^ Risk Score demonstrated no significant association with AF risk, with an AUC of 0.503 (*p* = 0.962), indicating a lack of discriminatory power in this context (Figure 4c).

### 3.5. Regional Analysis of FAI-Score Values and Total FAI Score

**Figure 5:**
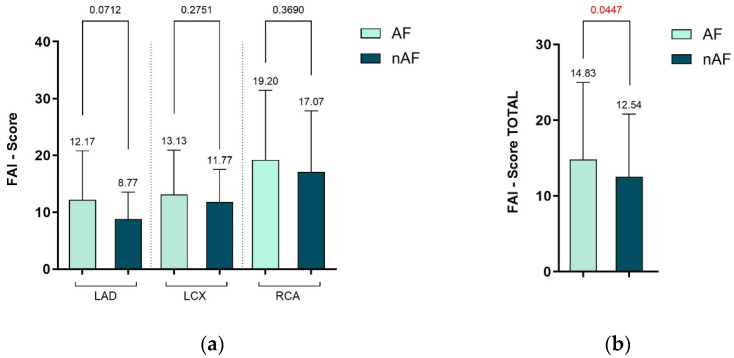
Regional analysis of (**a**) FAI score values and the (**b**) total FAI score, between the two study groups. Abbreviations: FAI–fat attenuation index.

As shown in Figure 5, we separately compared the FAI score for each of the three major coronary arteries between the two study groups. No significant differences were observed in the regional analyses. However, when evaluating the total FAI score, patients with AF had a significantly higher mean value compared to those without AF (14.83 vs. 12.54), with a *p*-value of 0.0447, indicating statistical significance.

When we categorized the FAI score percentiles into the following groups, ≤24.9th percentile (low), 25–49.9th percentile (minimal), 50–74.9th percentile (medium), 75–89.9th percentile (high), and ≥90th percentile (very high), we used these categories to stratify the study population accordingly. We observed a notable pattern: the majority of individuals in the non-AF group fell into the low-risk category, while those in the AF group were more frequently assigned to the higher-risk categories. A statistically significant difference was found in the minimal category, with a greater proportion of non-AF individuals compared to those with AF (31.25% vs. 9.52%, *p* = 0.0073). Conversely, in the very high category, the proportion of AF patients was significantly greater than that of non-AF individuals (33.33% vs. 13.75%, *p* = 0.0172) (Table 4).

## 4. Discussion

The principal findings of this study are summarized as follows: (1) Increased EAT volume is significantly associated with the presence of AF; (2) pericoronary inflammation quantified by FAI value is significantly associated with AF; (3) according to CAD-RADS classification, a lower degree of stenosis indicates lower prevalence of AF; (4) EAT, the FAI, and CAD-RADS provide valuable non-invasive imaging markers for AF risk assessment.

This study builds on our previous work in a distinct patient cohort [9], which showed similar per-vessel FAI trends, with significance reached only at the LAD level. In both investigations, total FAI scores were significantly elevated in AF patients, reinforcing its role as a more consistent and robust marker of coronary inflammation in the context of AF. Pericoronary and peri-atrial tissue characteristics, as assessed through imaging modalities such as CCT may reflect the inflammatory status of cardiac structures. This inflammation is associated with the onset and progression of AF and other cardiovascular diseases. Therefore, these tissue features could serve as potential markers of cardiovascular with predictive value for further events.

Gaibazzi et al. demonstrated that the volumetric attenuation of adipose tissue located posterior to the left atrium (LA) is independently associated with the presence of AF [21]. In our study, we analyzed the thickness of the EAT surrounding the LA, as well as bi-atrial EAT; however, the specific localization of EAT was not correlated with the presence of AF. Notably, patients with a documented history of AF exhibited a significantly higher mean volume of EAT. The interplay between EAT and AF appears to be bidirectional. While increased EAT volume and elevated FAI scores were strongly associated with AF in our study, the causal direction remains unclear. EAT may contribute to AF development by promoting local inflammation, fibrosis, and atrial remodeling—mechanisms supported by both clinical and preclinical studies [4,22,23]. On the other hand, AF itself may lead to structural and perfusional changes that stimulate further EAT accumulation and inflammatory activation. As our study was retrospective, we cannot determine causality. Prospective longitudinal research, including serial imaging before and after interventions such as catheter ablation, will be essential to clarify this relationship.

Furthermore, in our extended analysis, we included two key structural parameters—LAV and LAVI—both derived from contrast-enhanced CT scans. These metrics were significantly elevated in the AF group, confirming their established role as markers of atrial remodeling and predictors of arrhythmia risk [14,24,25,26]. While both LAV (AUC = 0.713) and the LAVI (AUC = 0.756) demonstrated independent predictive value, our findings indicate that EAT volume showed superior discriminative performance (AUC = 0.869). However, the FAI score (AUC = 0.562) did not outperform LAV, the LAVI, or EAT volume, and approached statistical significance (*p* = 0.0634). We chose to report both LAV and LAVI results due to their complementary value in characterizing atrial structural changes, and to ensure consistency with the existing literature. Notably, a combined score incorporating EAT volume and the LAVI yielded the highest discriminative performance (AUC = 0.887, sensitivity 92.86%, specificity 78.75%). However, formal statistical comparison using the DeLong test showed that the improvement over EAT alone was not significant (*p* = 0.186), suggesting that while the combined model offers slight numerical gains, the added predictive value is modest.

Similarly to the previous findings, Batal et al. reported a strong correlation between the posterior localization of adipose tissue adjacent to the left atrium and a higher burden of AF. Interestingly, posterior pericardiectomy during cardiac surgery has been linked to a reduced risk of postoperative AF [27]. This suggests that targeting fat deposits, particularly in the posterior region, may be relevant in mitigating AF risk and improving outcomes in affected patients. They further suggested that not only epicardial fat but also subcutaneous fat depots, obesity, BMI, age, and C-reactive protein level (CRP)—a marker of systemic inflammation—are linked to AF. Owing to the homogeneity of our study population, demographic parameters like age, obesity, and BMI did not correlate with the prevalence of AF; however, there are several studies in the literature demonstrating that EAT inflammation and its effects are independent from different clinical variables [28,29,30]. Although BMI did not differ significantly between groups, a weak but statistically significant correlation between BMI and EAT volume was observed only in AF patients. This suggests that EAT accumulation in AF may not solely reflect systemic obesity, but instead may involve local mechanisms such as inflammation and atrial remodeling. These findings support previous studies describing EAT as a regionally active fat depot with distinct metabolic and pro-arrhythmic properties [31,32].

In our study population, CACss were significantly higher in patients without AF. While initially surprising, this observation is consistent with growing evidence suggesting that extensive coronary calcification may reflect a more stable, chronic phase of CAD, often characterized by lower inflammatory activity. In contrast, patients with AF showed lower CACss but significantly elevated markers of inflammation and atrial remodeling—including the FAI, total EAT volume, and the LAVI—pointing to a more active, non-calcified disease phenotype [30,31,33]. This apparent paradox highlights the notion that pericoronary inflammation, rather than the burden of calcified plaque, may play a more decisive role in AF pathogenesis. Based on our results CADs had an inverse predictive performance. These findings are in line with previous studies demonstrating that inflamed coronary segments identified by the FAI can predict cardiovascular events even in patients with a low or zero CACs [18,34].

The literature findings indicate that EAT volume correlates with CAD severity and may serve as a predictor of disease progression [35]. In our study, EAT volume was associated with the burden of AF, whereas CAD-RADS categories did not show a statistically significant difference between patients with and without AF. This may further support the hypothesis that inflammation is a primary and early contributor to the development of CAD and related complications. Moreover, certain clinical trials have demonstrated that excessive EAT can contribute to the development of atherosclerosis by activating inflammatory pathways [36,37]. The metabolic activity of EAT is assessed by calculating the FAI, which, in our study, would be essential for providing more accurate and robust evidence of its correlation with AF.

## 5. Study Limitation and Possible Bias

One significant limitation of this study is its retrospective design, which limits the ability to infer causality between increased EAT, an elevated FAI score, and the presence of AF. It remains unclear whether excess pericardial fat promotes AF through local inflammation and atrial remodeling, or whether AF itself induces secondary changes in fat distribution and inflammatory activity. Future prospective studies—particularly involving patients undergoing catheter ablation with repeated CCT follow-up—will be essential to clarify this bidirectional relationship.

The retrospective approach was primarily driven by the high complexity and cost associated with inflammation-focused imaging workflows. This included secure anonymization, image export, and the cloud-based transfer of CT datasets to external AI platforms such as CaRi-Heart^®^. These technical and logistical challenges limited the feasibility of a prospective longitudinal design at the current stage.

Another notable limitation is the relatively small sample size, particularly within the AF subgroup. This may have reduced the statistical power to detect subtle associations between FAI score and AF risk, especially where borderline significance was observed. The modest cohort size reflects the strict inclusion criteria and the technical intensity of full FAI analysis. Each scan required meticulous anonymization, upload, and processing through advanced AI-driven radiomic software—a time- and resource-intensive process.

Additionally, although we incorporated LAV and the LAVI into our analysis, echocardiographic validation was not performed, and longitudinal atrial remodeling over time could not be assessed. Future studies should consider multimodality imaging to strengthen structural interpretation.

Detailed pharmacological data were not systematically analyzed, as the primary focus of the study was on imaging-derived inflammatory markers. However, due to the relative homogeneity of comorbidities across the study groups, significant differences in anti-inflammatory medication use were unlikely. Additionally, systemic inflammatory biomarkers such as CRP or interleukin-6 were not routinely available for all patients, limiting our ability to directly correlate plasma-based and imaging-based measures of inflammation.

These limitations underscore the need for multicenter collaborations and streamlined imaging platforms to facilitate broader, large-scale implementation of inflammation-based cardiac risk stratification.

## 6. Conclusions

Personalized risk stratification for AF is increasingly feasible through the integration of artificial intelligence and advanced imaging biomarkers. Non-invasive parameters such as EAT volume, FAI score, CAD-RADS classification, and the LAVI collectively enhance our ability to identify individuals who are at elevated risk for AF. In patients currently in sinus rhythm, elevated EAT and FAI values may serve as early warning signs for future AF development, supporting closer monitoring and more aggressive risk factor control. This multidimensional approach—combining inflammatory, structural, and anatomical CT-derived metrics—holds promise for guiding early screening and tailoring preventive strategies. It may also serve as a foundation for developing more targeted, preemptive therapeutic interventions in the future.

## Figures and Tables

**Table 1:** Baseline characteristics, comorbidities, and risk factors in the study population.

Parameters	Whole Study Sample (*n* = 122)	Group 1 (Patients with AF) (*n* = 42)	Group 2 (Patients without AF) (*n* = 80)	*p* Value
Age, mean ± SD	63.70 ± 7.78	64.05 ± 7.84	63.53 ± 7.78	0.6628
Male gender, *n* (%)	75 (61.47%)	25 (59.52%)	50 (62.50%)	0.8452
BMI ^1^, (kg/m^2^), mean ± SD	27.44 ± 4.32	28.25 ± 5.07	27.05 ± 3.86	0.2567
Cardiovascular risk factors:				
Hypertension, *n* (%)	75 (61.47%)	29 (69.05%)	46 (57.50%)	0.2441
Hypercholesterolemia, *n* (%)	43 (35.24%)	17 (40.48%)	26 (32.50%)	0.4278
Diabetes, *n* (%)	32 (26.22%)	10 (23.81%)	22 (27.50%)	0.8288
Smoking, *n* (%)	27 (22.13%)	11 (26.19%)	16 (20.00%)	0.4937

^1^ BMI—Body Mass Index.

**Table 2:** CCT imaging biomarkers: LAVI, EAT, CACs, and CAD-RADS in AF evaluation.

Parameters	Whole Study Sample (*n* = 122)	Group 1 (Patients with AF) (*n* = 42)	Group 2 (Patients without AF) (*n* = 80)	*p* Value
LAV ^1^ (mL), mean ± SD	92.15 ± 23.45	103.3 ± 20.34	86.3 ± 22.94	<0.0001
LAVI ^2^ (mL/m^2^), mean ± SD	49.96 ± 13.39	57.7 ± 11.44	45.9 ± 12.58	<0.0001
EAT ^3^ assessment:				
EAT volume (mL), mean ± SD	180.3 ± 63.5	231.8 ± 45.85	153.2 ± 54.14	<0.0001
EAT-LA ^4^ volume (mL), mean ± SD	18.30 ± 8.7	23.55 ± 6.44	15.54 ± 8.49	<0.0001
EAT-BA ^5^ volume (mL), mean ± SD	43.42 ± 15.5	50.24 ± 12.69	39.84 ± 15.70	0.0002
CACs ^6^ extent, mean ± SD:	378.2 ± 137.1	231.9 ± 76.98	455.1 ± 91.27	<0.0001
CACs < 10, *n* (%)	70 (57.37%)	28 (66.67%)	42 (52.50%)	0.1775
CACs: 10–400, *n* (%)	31 (25.40%)	8 (19.05%)	23 (28.75%)	0.2800
CACs > 400, *n* (%)	21 (17.21%)	6 (14.29%)	15 (18.75%)	0.6202
CAD-RADS ^7^ categories:				
0–1, *n* (%)	49 (40.16%)	11 (26.19%)	38 (47.75%)	0.0320
2–3, *n* (%)	42 (34.42%)	17 (40.48%)	25 (31.25%)	0.3230
4–5, *n* (%)	31 (25.40%)	14 (33.33%)	17 (21.25%)	0.1892

^1^ LAV—Left Atrial Volume; ^2^ LAVI—Left Atrial Volume Index; ^3^ EAT—Epicardial Adipose Tissue, ^4^ EAT-LA—Epicardial Adipose Tissue at the level of the Left Atrium; ^5^ EAT-BA—Epicardial Adipose Tissue at the Bi-atrial level; ^6^ CACs—Coronary Artery Calcium Score; ^7^ CAD-RADS—Coronary Artery Disease-Reporting and Data System.

**Table 3:** ROC curve analysis for CCT imaging markers in correlation with AF and non-AF patients.

Parameters	AUC ^1^	95% CI for AUC	z-Statistic	Youden Index	Cu-Off Value for AF	Sensitivity (%)	Specificity (%)	*p* Value
LAV ^2^ (mL), mean ± SD	0.713	0.62–0.79	4.43	0.37	>92.72	73.81	63.75	<0.0001
LAVI ^3^ (mL/m^2^), mean ± SD	0.756	0.67–0.82	5.75	0.45	>52.25	73.81	71.25	<0.0001
EAT ^4^ volume, mL	0.869	0.79–0.92	11.40	0.66	>179.01	92.86	73.75	<0.0001
EAT + LAVI (combined score)	0.887	0.81–0.93	12.76	0.71	>232.58	92.86	78.75	<0.0001
EAT-LA ^5^ volume, mL	0.776	0.69–0.84	6.61	0.44	>17.74	83.33	61.25	<0.0001
EAT-BA ^6^ volume, mL	0.703	0.61–0.78	4.09	0.33	>49.95	57.14	76.25	<0.0001
CACs ^7^ extent	0.968	0.91–0.99	35.31	0.83	≤352.21	97.62	86.25	<0.0001
FAI ^8^—Score _TOTAL_	0.562	0.50–0.61	1.85	0.18	>18.49	37.30	81.25	0.0634
CaRi-Heart^®^ risk score	0.503	0.41–0.59	0.04	0.07	≤ 13.16	30.95	61.25	0.9622

^1^ AUC—Area Under the Curve; ^2^ LAV—Left Atrial Volume; ^3^ LAVI—Left Atrial Volume Index; ^4^ EAT—Epicardial Adipose Tissue; ^5^ EAT-LA—Epicardial Adipose Tissue at the level of the Left Atrium; ^6^ EAT-BA—Epicardial Adipose Tissue at the Bi-atrial level; ^7^ CACs—Coronary Artery Calcium Score; ^8^ FAI—Fat Attenuation Index.

**Table 4:** PCAT-FAI scores, FAI score percentile categories and the CaRi-Heart^®^ Risk Score for the study population.

Parameters	Whole Study Sample (*n* = 122)	Group 1 (Patients with AF) (*n* = 42)	Group 2 (Patients without AF) (*n* = 80)	*p* Value
FAI ^1^ scores:				
FAI—Score _LAD_, mean ± SD	9.69 ± 5.94	12.17 ± 8.61	8.77 ± 4.78	0.0712
FAI—Score _LCX_, mean ± SD	12.24 ± 6.51	13.13 ± 7.78	11.77 ± 5.73	0.2751
FAI—Score _RCA_, mean ± SD	17.80 ± 11.29	19.20 ± 12.25	17.07 ± 10.77	0.3690
FAI—Score _TOTAL_, mean ± SD	13.04 ± 8.49	14.83 ± 10.16	12.37 ± 7.89	0.0447
CaRi-Heart^®^ risk score, mean ± SD	18.92 ± 12.5	19.12 ± 12.84	18.81 ± 12.40	0.8647
FAI—Score Centile categories:				
≤24.9th percentile (low)	11 (9.01%)	2 (4.76%)	9 (11.25%)	0.3273
25–49.9th percentile (minimal)	29 (23.77%)	4 (9.52%)	25 (31.25%)	0.0073
50–74.9th percentile (medium)	30 (24.59%)	9 (21.43%)	21 (26.25%)	0.6604
75–89.9th percentile (high)	27 (22.13%)	13 (30.95%)	14 (17.50%)	0.1096
≥90th percentile (very high)	25 (20.49%)	14 (33.33%)	11 (13.75%)	0.0172

^1^ Abbreviations: FAI—Fat Attenuation Index; LAD—Left Anterior Descending artery; LCX—Left Circumflex artery; RCA—Right Coronary Artery.

## Data Availability

The data presented in this study are available upon request from the corresponding author. The data are not publicly available due to privacy reasons.

## Abbreviations

The following abbreviations are used in this manuscript:

AF	Atrial Fibrillation
AUC	Area Under the Curve (ROC analysis)
EAT	Epicardial Adipose Tissue
EAT-LA	Epicardial Adipose Tissue at the level of the Left Atrium
EAT-BA	Epicardial Adipose Tissue at the Bi-atrial level
LAV	Left Atrial Volume
LAVI	Left Atrial Volume Index
CCT	Cardiac Computed Tomography
FAI	Fat Attenuation Index
FAI-HU	Fat Attenuation Index measured in Hounsfield Units
CAD	Coronary Artery Disease
CAD-RADS	Coronary Artery Disease-Reporting and Data System
PCAT	Pericoronary Adipose Tissue
PCAT-FAI	Fat Attenuation Index of Pericoronary Adipose Tissue
CACs	Coronary Artery Calcium Score
HU	Hounsfield Units
ROC	Receiver Operating Characteristic
LAD	Left Anterior Descending artery
LCX	Left Circumflex artery
RCA	Right Coronary Artery
BMI	Body Mass Index
CRP	C-Reactive Protein
